# Magnetic resonance features of the feline hippocampus in epileptic and non-epileptic cats: a blinded, retrospective, multi-observer study

**DOI:** 10.1186/s12917-016-0788-3

**Published:** 2016-08-11

**Authors:** Anne Christine Claßen, Sibylle Kneissl, Johann Lang, Alexander Tichy, Akos Pakozdy

**Affiliations:** 1 Small Animal Veterinary Hospital Stommeln, D 50259 Pulheim, Germany; 2 Clinical Unit of Diagnostic Imaging, University of Veterinary Medicine, A 1210 Vienna, Austria; 3 Division of Clinical Radiology, Vetsuisse Faculty, University of Bern, CH 3012 Bern, Switzerland; 4 Department of Biomedical Sciences, University of Veterinary Medicine, A 1210 Vienna, Austria; 5 Clinic for Internal Medicine, University of Veterinary Medicine, A 1210 Vienna, Austria

**Keywords:** Feline temporal lobe epilepsy, Orofacial automatism, Orofacial involvement, Magnetic resonance imaging, Epileptic seizure, Hippocampal necrosis, Hippocampal sclerosis, Interobserver agreement, Cluster seizures, Status epilepticus

## Abstract

**Background:**

Hippocampal necrosis in cats has been reported to be associated with epileptic seizures. Magnetic resonance imaging (MRI) features of temporal lobe (TL) abnormalities in epileptic cats have been described but MR images from epileptic and non-epileptic individuals have not yet been systematically compared. TL abnormalities are highly variable in shape, size and signal, and therefore may lead to varying evaluations by different specialists. The aim of this study was to investigate whether there were differences in the appearance of the TL between epileptic and non-epileptic cats, and whether there were any relationships between TL abnormalities and seizure semiologies or other clinical findings. We also investigated interobserver agreement among three specialists.

**Methods:**

The MR images of 46 cats were reviewed independently by three observers, who were blinded to patient data, examination findings and the review of the other observers. Images were evaluated using a multiparametric scoring system developed for this study. Mann–Whitney U-tests and chi-square were used to analyse the differences between observers’ evaluations. The kappa coefficient (k) and Fleiss’ kappa coefficient were used to quantify interobserver agreement.

**Results:**

The overall interobserver agreement was moderate to good (k =0.405 to 0.615). The MR scores between epileptic and non-epileptic cats did not differ significantly. However, there was a significant difference between the MR scores of epileptic cats with and without orofacial involvement according to all three observers. Likewise, MR scores of cats with cluster seizures were higher than those of cats without clusters.

**Conclusion:**

Cats presenting with recurrent epileptic seizures with orofacial involvement are more likely to have hippocampal pathologies, which suggests that TL abnormalities are not merely unspecific epileptic findings, but are associated with a certain type of epilepsy. TL signal alterations are more likely to be detected on FLAIR sequences. In contrast to severe changes in the TL which were described similarly among specialists, mild TL abnormalities may be difficult to interpret, thus leading to different assessments among observers.

## Background

Over the past two decades, several case reports on feline hippocampal necrosis (HN) have been published in Europe, Asia, and the United States [1–9], indicating that necrosis of the hippocampus and piriform lobe associated with the appearance of epileptic seizures in feline patients is a worldwide phenomenon. Magnetic resonance imaging (MRI) is routinely used in human epileptic patients [10–12], and hippocampal sclerosis (HS) has been identified as the single most frequently detected feature of mesial temporal lobe (TL) epilepsy in humans [11].

In cats, MRI is also the method of choice for ante-mortem detection of intracranial pathologies, including hippocampal changes. However, there has not been a systematic comparison of MR images of TLs in healthy and epileptic cats, and little is known about the accuracy of this diagnostic modality or the potential subjectivity of the observers’ assessments. Thus, it remains unclear whether changes in the hippocampal size and signal are always associated with the occurrence of epileptic seizures or whether they can also be present in non-epileptic patients.

The aims of this retrospective, multi-observer study were to examine whether there was a difference in the TL signal between epileptic and non-epileptic cats and whether an abnormal MR signal was related to seizure semiology or other clinical findings in epileptic patients. In addition, the degree of agreement among three independent observers was assessed. We hypothesised that marked hippocampal signal alterations in MRI would be associated with the occurrence of epileptic seizures in feline patients. Cats suffering from TL seizures (including orofacial automatisms) were expected to be diagnosed with hippocampal lesions more often than cats suffering from non-temporal epileptic seizures of different aetiologies. We also expected good agreement among specialists in identifying signal alterations, and that fluid-attenuated inversion recovery (FLAIR) sequences would be superior to other MR sequences in detecting variations in TL signal intensity.

## Methods

Medical records of cats housed at the University of Veterinary Medicine, Vienna, between August 2011 and May 2013 were searched for animals that underwent high field-MRI (Magnetom Espree, 1.5 Tesla, Siemens Healthcare, Erlangen, Germany). Inclusion criteria were availability of at least one transverse T1-weighted scan, one post-contrast T1 turbo spin-echo (T1C), one FLAIR, and either one transverse T2 or 3D sagittal T2-weighted scan. A series of subtracted pre- and post-contrast images was found for most cases. Imaging studies that met the inclusion criteria were selected independently by A.C., who was not actively involved in the review process. All patients were anonymised by assigning a randomised patient number.

The images were independently evaluated by one neurologist (AP, DECVN) and two radiologists (SK, non-certified; JL, DECVDI). Each of the three observers had more than 10 years of experience in MRI interpretation. The observers were blinded to patient data and history, examination findings, and the review of the other two observers.

Observers recorded their evaluations on the TL signal using an itemised form. The multiparametric scoring system consisted of two subdivisions (Code I, II Table 1). Code I classified the signal intensity of the hippocampi in relation to the paramedian grey matter; the normal hippocampal signal intensity was considered to be slightly hyperintense on FLAIR and T2-weighted images compared to the paramedian grey matter. Code II consisted of signal intensity, signal shape, and contrast enhancement as parameters, which were combined into a total score (overall assessment); this code was recently used to describe MR images in dogs [13] and was modified for this study.

**Table 1:** Scoring scheme used according to codes I and II

Score	Code I	Code II	
1	Normal	Definitely normal	Normal TL signal, shape and no contrast enhancement
2	Questionable	Probably normal	Slightly increased/decreased TL signal
3	Moderately increased or decreased signal intensity	Equivocal	Distinctly increased/decreased TL signal
4	Severely increased or decreased signal intensity	Probably abnormal	Distinctly increased/decreased TL signal and abnormal shape
5	-	Definitely abnormal	Abnormal TL signal, shape and contrast enhancement

Code I - Hippocampal signal intensity

Code II - Overall TL signal and shape characteristics

### Statistical analyses

The statistical analyses were performed using IBM SPSS v. 20 (SPSS Inc., Chicago, IL, USA).

A Mann–Whitney *U*-test was used to analyse the differences in TL MR morphology between patients grouped by history, that is, evidence of epileptic seizures, status epilepticus (SE), cluster seizures (CS), orofacial involvement (OI), and number of epileptic seizures prior to MRI. Status epilepticus was defined as seizures of more than thirty minutes duration or repeated seizures without full recovery whereas cluster seizures were determined as more than one seizure within 24 h.

The weighted kappa coefficient (k) was used to quantify pairwise agreement between observers. A value of 1.0 implied complete agreement and 0.0 indicated a level of agreement expected by chance alone (categories were: 0.0–0.2 poor agreement; 0.21–0.40 fair agreement; 0.41–0.60 moderate agreement; 0.61–0.80 good agreement; 0.81–1.0 excellent agreement). Fleiss’ kappa coefficient for multiple raters was also calculated to estimate the overall agreement among the three observers. For all statistical tests, *p* < 0.05 was considered significant.

An additional system was introduced to compare the individual overall assessments of each observer. A four-point gap between scores was considered poor agreement, three points indicated fair agreement, two points showed moderate agreement, and a one point difference indicated good agreement. The agreement was considered perfect if the observers rated the images with the same score.

## Results

MR images of 46 cats were included in the study. Twenty-five cats (54.3 %) were females (22 spayed and three intact) and 21 (45.7 %) were males (20 neutered and one intact). The mean age of the cats was 7 years (range 3 months to 15 years), and the breeds represented were European shorthair (*n* = 32), domestic shorthair (*n* = 8), and one of each of the following breeds: Persian, Norwegian forest cat, Maine Coon, Devon rex, European longhair, and Chartreux.

The indication for brain MRI was epileptic seizures in 27 cases (58.7 %) and other neurological disorders in 19 (41.3 %) cats. Nineteen epileptic cats (41.3 %) showed CS and seven (15.2 %) had experienced at least one SE. Fifteen cats (32.6 %) presented epileptic seizures with OI. Of these, eight (17.4 %) had 1–5 seizures, five (10.9 %) had 6–10, five (10.9 %) had 11–25, and eight (17.4 %) had > 25 epileptic fits prior to undergoing MRI. In one case (2.2 %), it was not possible to clarify how many seizures the animal had experienced prior to MRI because it was owned by an animal shelter.

Four of the nineteen non-epileptic patients presented with tetraparesis (8.7 %), three presented with vestibular dysfunction (6.5 %), and two cats with each of circling (4.3 %), obsessive walking (4.3 %), aggression (4.3 %), or anisocoria (4.3 %). One cat each was examined because of hemiparesis (2.2 %), collapse (2.2 %), behavioural changes other than aggression (2.2 %), and for medical evaluation of cerebellar rigidity (2.2 %). MRI of the non-epileptic patients revealed intracranial neoplasia (31,6 %), unremarkable MRI scan (31,6 %), extracranial neoplasia (21,1 %), internal otitis (10,5 %) and ear polyp (5,3 %).

Using the additional system of testing agreement by comparing the magnitude difference between scores, the overall agreement was perfect among three observers in 52.2 % of cases and perfect to good (i.e., maximum difference of one point between scores) in 67.4 % cases. In 17.4 % of cases the overall agreement was fair to poor. The overall interobserver agreement (Code II) ranged from k = 0.405 to 0.615. Of all comparisons between observers, observers A and B had the best agreement, with a k value of 0.615. All interobserver analyses resulted in 0.8 > k >0.4, which is considered to be moderate to good agreement. Fleiss’ kappa was 0.493, which also implies a moderate agreement among all three observers.

Table 2 lists the mean MR scores (regarding the overall assessment, Code II) for TL MR morphology for patients with different histories. For statistical analysis rating distributions were compared using chi-square test. The rating distribution did not differ between epileptic and non-epileptic cats (observers A *p* = 0.27, B *p* = 0.283, C *p* = 0.528; overall *p* = 0.111).

**Table 2:** Mean MR scores (Code II) from observers A, B, and C for patients with different histories. For statistical analysis chi-squared comparison of rating distribution was used

	Observer		
Patient History	A	B	C
Epileptic cats	2.3	2.1	2.5
Nonepileptic cats	1.4	1.6	1.8
Epileptic cats without orofacial involvement	1.1	1.2	1.4
Epileptic cats with orofacial involvement	3.2	2.9	3.4
Epileptic cats with status epilepticus	3.3	3.1	3.3
Epileptic cats without status epilepticus	1.7	1.6	2.1
Epileptic cats with cluster seizures	2.6	2.3	3.2
Epileptic cats without cluster seizures	1.4	1.5	1.6
Cats with 1–5 seizures prior to MRI	1.9	1.8	1.4
Cats with 6–10 seizures prior to MRI	1.0	1.0	1.3
Cats with 11–25 seizures prior to MRI	2.8	2.3	4.2
Cats with >25 seizures prior to MRI	2.9	2.8	3.1

The MR images of cats that had a history of epileptic seizures with OI were rated with mean Code II scores of 3.2 (observer A), 2.9 (observer B), and 3.4 (observer C). The rating distribution of epileptic cats with OI differed significantly from epileptic cats without OI (observer A: *p* < 0.001; observer B:, *p* =0.002; observer C:, *p* = 0.027, overall *p* = 0.001) and from the non-epileptic cats (observer A:, *p* = 0.009; observer B:, *p* = 0.071; observer C: *p* = 0.228, overall: *p* = 0.001).

**Fig. 1:**
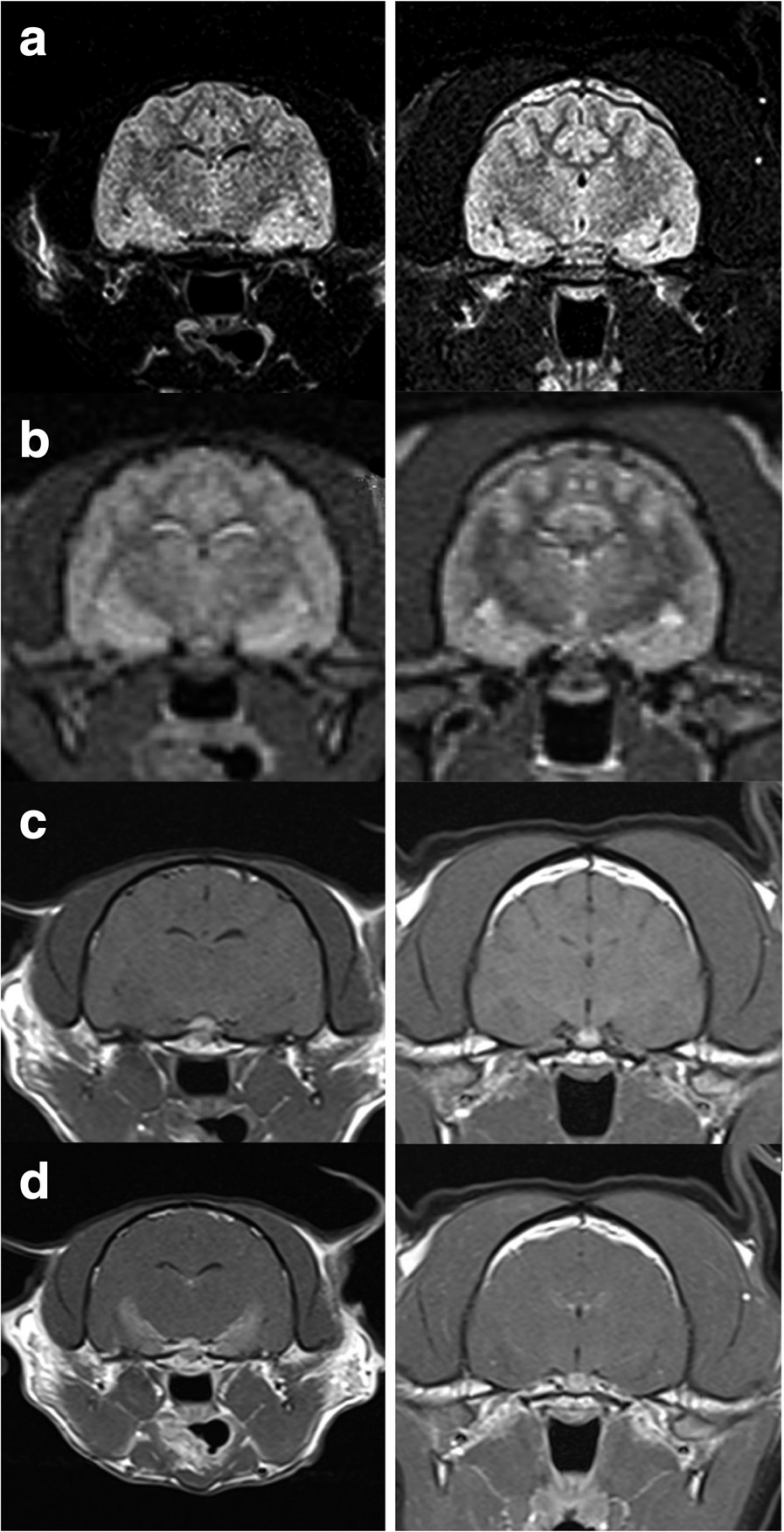
Different MR sequences at the level of the hippocampus and piriform lobe of two cats for comparison. Transverse FLAIR (**a**), T2-weighted (**b**), pre- (**c**) and post-contrast (**d**) T1-weighted images at the level of the hippocampus and piriform lobe in a 9-year-old male Maine Coon with reported seizures with orofacial involvement (*left*) and 3-year-old female neutered European Shorthair Cat suffering from epileptic seizures without orofacial automatisms (*right*). The MR overall assessment (Code II) was considered definitely abnormal (score 5) by all three observers in the former and definitely normal (score 1) in the latter case

The TL MR morphologies of epileptic cats with at least one SE were rated significantly higher than those that had never experienced SE only by observers A (observer A: *p* = 0.016; observer B: *p* = 0.067; observer C: *p* = 0.376, overall: *p* = 0.001). In addition, all observers rated the images of cats with a history of CS with a significantly higher Code II score than cats with no history of CS (observer A: *p* = 0.012; observer B: *p* = 0.076; observer C: *p* = 0.0028; overall *p* < 0.001 Fig. 1).

There was a tendency that with an increasing number of seizures the TL abnormalities were more severe, however the difference was not significant. Significant and nearly significant was the difference regarding Code II scores given for images of the subgroup of epileptic cats that had 11–25/more than 25 seizures compared to non-epileptic cats (A: *p* = 0.066/0.083, B: *p* = 0.23/0.096, C: 0.092/0.321, overall 0.009/0.051).

Table 3 lists the mean Code I scores for the hippocampal signal intensity changes seen on the five MR sequences evaluated. Higher Code I scores (scores range from 1–4 points) illustrate greater signal changes of the hippocampal signal. The mean of Code I scoring points given for T2 images was 1.5 (observer A), 1.7 (observer B), and 1.8 (observer C), which referred to a normal to questionable signal intensity of the hippocampi. The mean Code I scores given for the FLAIR images were 2.0 (observer A), 1.7 (observer B), and 1.9 (observer C). These results referred to a normal to questionable signal intensity (observers B and C) or questionable signal intensity (observer A). The signal intensity of native T1-weighted images was given a mean score of 1.4 (observer A), 1.5 (observer B), and 1.0 (observer C). These results referred to a normal (observer C) or normal to questionable signal intensity (observers A and B). The T1-weighted images after intravenous contrast application were given mean Code I scores of 1.6 (observer A), 1.4 (observer B), and 1.6 (observer C), all normal to questionable signal intensities. Finally, the subtracted pre- and post-contrast images were given mean scores of 1.4 (observer A), 1.4 (observer B), and 1.7 (observer C), all normal to questionable signal intensities.

**Table 3:** Comparison of MR assessment of signal change (Code I) using different sequences

	Observer A				Observer B				Observer C			
SP	1	2	3	4	1	2	3	4	1	2	3	4
T2	35	2	8	1	30	3	8	4	22	11	10	1
	76.1	4.3	17.4	2.2	66.7	6.7	17.8	8.9	50.0	25.0	22.7	2.3
FLAIR	24	2	15	5	30	3	7	4	21	10	11	2
	52.2	4.3	32.6	10.9	68.2	6.8	15.9	9.1	47.7	22.7	25.0	4.5
T1	38	0	5	2	32	4	8	1	43	0	1	0
	84.4	0.0	11.1	4.4	71.1	8.9	17.8	2.2	97.7	0.0	2.3	0.0
T1C	35	0	6	5	37	0	3	4	30	4	6	4
	76.1	0.0	13.0	10.9	84.1	0.0	6.8	9.1	68.2	9.1	13.6	9.1
SUB	35	0.0	3	4	35	2	1	4	27	1	6	4
	83.3	0.0	7.1	9.5	83.3	4.8	2.4	9.5	71.1	2.6	15.8	10.5

SP: MR scoring points, where 1 = normal signal, 2 = questionable, 3 = slightly increased or decreased, and 4 = severely increased or decreased. Table shows counts in first row and percentages in second row

## Discussion

As a group, MR images of the TL of cats suffering from any kind of epileptic seizure did not differ significantly from those of cats without epileptic disorders. However when stratified according to seizure semiology, or occurrence of CS or SE, MR images of the TL of cats with a history of epileptic seizures with OI, or cats with CS or SE, differed significantly from epileptic cats that did not, and from non-epileptic cats.

Previous studies have suggested that alterations in the feline TL visible on MR images were caused by various underlying conditions, including astrogliosis, oedema, hypoxia, idiopathic HN and HS, inflammation, intracranial infection, ischemia, malformation and neoplastic conditions [1–9]. A potential toxin or infectious agent cannot be ruled out, nor can genetic predisposition or febrile seizures early in life, as described in human medicine [14]. The hippocampus is also known to have a very low seizure threshold compared to other parts of the brain and is therefore more susceptible to active participation in a post-discharge evoked by a different part of the brain, or even to become an epileptic focus itself [15]. Since the initial histological description of HS in humans, there has been debate about whether HS is a nonspecific result of a primary epileptogenic lesion restricted to the hippocampus, whether it is caused by damage to the cells due to the spread of epileptic discharges to this area, or even whether it develops due to other causes [11]. There is evidence that HN and HS in children is multi-causal [16]; the same can be reasonably assumed for feline patients. At present it is not possible to distinguish the underlying cause of a TL abnormality by diagnostic imaging alone, even if repeated scans could help to differentiate between reversible and irreversible lesions.

Changes in the TL MR morphology seem to be rare in non-epileptic cats and occur much more frequently in epileptic cats with OI compared to other cats. Therefore, we suspect that OI in epileptic cats is not only a specific epileptic phenomenon for feline temporal lobe epilepsy (TLE), in line with early experimental studies [17], but is also significantly associated with temporal lobe changes detected in MR images.

As expected, the images of cats with a history of CS or SE were given significantly higher MR scores (however not by all three observers) compared to the images of cats that had never suffered CS or SE. These groups also included cats presenting OI, since the majority of the cats with OI also had CS or SE.

SE and CS likely cause more severe damage to neurons compared to infrequent short seizures due to the long period in which neurons are exposed to synchronous electric activity [18]. Previous studies have shown that the hippocampus, in particular, tends to develop irreversible MRI-detectable pathologies after convulsive SE [19]. However, we did not evaluate the time span between the last observed epileptic event and the day of diagnostic imaging. Therefore, it is possible that even if there was acute damage to the neurons of the TL, this damage could have been reversible and there may have been sufficient time for the cells to reorganise before the images were taken. This aspect has never been investigated in cats but should be taken into account in further studies as early postictal MRI abnormalities can also be both causes and consequences of seizure [18].

We expected that signal alterations in the hippocampal area would be detected more frequently in cats that experienced a higher number of seizures prior to MRI. Indeed, there was such a tendency but differences in ratings were only significant between non-epileptic patients and patients with 11–25 or more than 25 seizures prior to MRI. These results may indicate that a certain number of a specific kind of seizure is necessary to cause visible MRI changes. Similarly, recent studies in rats showed that hippocampal volume loss was not correlated with seizure frequency [20]. Since the certain number of seizures our subjects had experienced could only be estimated in some cases, these data must be interpreted with caution. Moreover, because the number of postictal days prior to imaging is unknown, MR abnormalities could also be caused by reversible oedema.

In our study, FLAIR sequences were rated with the highest scores. Because the signal of intraventricular cerebrospinal fluid is suppressed, the FLAIR sequence for periventricular lesions is more conspicuous than T2-weighted sequences. T2-weighted sequences were only slightly inferior to FLAIR sequences in detecting alterations of the TL signal, consistent with earlier results [21, 22]. T1C-weighted images were ranked third, and pre-contrast T1-weighted images were ranked fourth. This leads to the conclusion that only lesions that show contrast enhancement are reasonably detectable on T1-weighted images. In human medicine, the gold standard scan protocol includes high-resolution T2-weighted images, with or without inverting the contrast. MR techniques such as hippocampal volume measurements, T2 relaxometry, MR spectroscopy and diffusion tensor imaging of the hippocampus are sometimes used in human medicine [23]. These dedicated techniques are not yet routinely available in veterinary medicine and MR volumetry and diffusion tensor imaging were only performed on few cats experimentally [24, 25]. The hippocampal volume was significantly reduced unilaterally in one study in familial strain of spontaneous epileptic cats in comparison with a healthy control group [24], but this aspect was not investigated by our study.

In this study, there was moderate-good interobserver agreement among specialists, although the overall agreement was perfect among the three observers in 52.2 % of cases and perfect to moderate in 82.6 % of all cases. There were many factors influencing the assessment of images in this blind study. Because the hippocampal signal was not measured objectively in this study, MRI interpretation was associated with considerable subjectivity, although this aspect has not been explicitly discussed in veterinary medicine. Opinions differ in clinical practice regarding what constitutes a normal TL signal, and this discrepancy may result in markedly different assessments among observers [26]. Because the hippocampus contains more grey matter than surrounding parts of the brain [27], mild T2 hyperintensity should be considered normal, but no standard value can be given. Although all three observers involved in this study were expert in neuroimaging, they had diverse backgrounds and different numbers of years’ experience in this specific field of diagnostic imaging, which could have led to different interpretations of the lesion anatomic location, pattern, mass effect, and contrast medium uptake. This discrepancy may also be explained, at least in part, by the tendency of some practitioners to score as uncertain for equivocal patients, whereas others prefer to commit to a diagnosis. Because of partial volume averaging it also may be difficult to distinguish whether the hyperintense signal arises from the hippocampus or the lateral ventricle and in some cases there were intracranial masses present, which displaced the hippocampal tissue and may have interfered with the MR signal of this region. If this was the case, some observers refused to evaluate the specific images. We conclude that subjectivity of the assessments is also a factor that may lead to discrepant evaluations and thus must not be underestimated.

This study had several limitations. First, it was a descriptive study of the agreement among three observers. Because it did not take the final diagnosis into account (most of the patients are still alive and only few histopathologic examinations have been performed yet), no conclusion could be made to the comparability of the radiographic and pathohistologic assessments. Second, the observers had no knowledge of the patients’ histories or physical and neurological examinations and could not contextualise the images. Third, we can not rule out for sure, that patients presenting with altered mentation or behavioral changes were suffering from non-convulsive status epilepticus and were misclassified as non-epileptics. Fourth, although the images were all taken at the same institution, some variability in positioning and MR sequence availability were noted. Fifth, our observers had little specific training in using the scoring system provided and it is possible that this also had an effect on the scoring. Finally, because there was no follow-up imaging, observers could not differentiate between reversible and irreversible changes of the affected side. However, such differentiation is likely relevant but this issue was investigated in cats only experimentally [28]. Volumetry could be helpful as hippocampal size might increase in oedema and decreases in sclerosis. The recently published recommendation on a veterinary epilepsy-specific MRI protocol by the International Veterinary Epilepsy Task Force also emphasised the importance of the evaluation of the hippocampi. At least visual assessment should be carried out including size, symmetry, atrophy and signal changes, however volumetric measurement may be meaningful in the future [29].

## Conclusions

Temporal lobe epilepsy with hippocampal sclerosis is well researched in human medicine, and there is strong evidence that TLE with HS may also be common in cats. Feline patients presenting with recurrent epileptic seizures with orofacial involvement or cluster seizures/status epilepticus are more likely to have hippocampal pathologies that are detectable by MRI and especially on FLAIR sequences. Changes in temporal lobe signal intensity (especially T2 hyperintensity) are more pronounced in cats suffering from TLE than in cats with any other kind of epileptic disorders and are therefore most likely specific pathophysiologic phenomena of this condition. However, non-specific consequence from status epilepticus or cluster seizures may play a role.

Mild hippocampal signal alterations may be difficult to interpret and lead to different assessments among observers, in contrast to severe changes, which are described in a similar way by different specialists.

## Abbreviations

CS, cluster seizures; FLAIR, fluid attenuated inversion recovery; HN, hippocampal necrosis; HS, hippocampal sclerosis; MRI, magnetic resonance imaging; OI, orofacial involvement; SE, status epilepticus; SUB, subtracted pre- and post-contrast images; T, Tesla (unit of the magnetic field); T1, T1-weighted image; T1C, post-contrast T1-weighted image; T2, T2-weighted image; TL, temporal lobe; TLE, temporal lobe epilepsy
